# Editorial: Ketogenic metabolic therapies in prevention & treatment of non-communicable diseases

**DOI:** 10.3389/fnut.2026.1797644

**Published:** 2026-02-17

**Authors:** David Cavan, Johannes J. Kovarik, Peter J. Voshol

**Affiliations:** 1 University Hospitals Dorset NHS Foundation Trust, Bournemouth, United Kingdom; 2 Department of Internal Medicine III Clinical Division of Nephrology and Dialysis, Medical University of Vienna, Vienna, Austria; 3 Independent Researcher, Culemborg, Netherlands

**Keywords:** cellular energy homeostasis, deprescription, epigenetic modulation, Ketogenic Metabolic Therapy, metabolic flexibility, neuro-inflammation, nutritional ketosis, transdiagnostic remission

## A wondering mind on the metabolic shift

Many of the chronic diseases that have traditionally been regarded as unavoidable features of aging may instead reflect maladaptive physiological responses arising from a mismatch between contemporary environmental exposures, nutrition and evolutionarily conserved metabolic regulatory systems. In considering the 19 articles included in this Research Topic, one is left with a sense of scientific curiosity and re-evaluation of prevailing models. Collectively, these works illustrate a transition away from a glucose-dominant framework toward one that seeks to restore the organism's inherent metabolic flexibility, characterized by the capacity to alternate efficiently between carbohydrate- and lipid-derived fuels.

For a long time, we viewed the ketogenic diet as a niche tool for epilepsy. However, the evidence presented here suggests that Ketogenic Metabolic Therapy (KMT) is a systemic intervention that re-tunes the body's fundamental signaling pathways. We find ourselves asking: how deep does this metabolic rabbit hole go?

## The gut as the metabolic gateway

Perhaps the most provocative question raised in this volume concerns the gut. In our traditional view, a healthy gut requires high fiber and diverse plant intake. Yet, the case series by Norwitz and Soto-Mota regarding a carnivore-ketogenic diet for Inflammatory Bowel Disease (IBD) invites us to pause. How is it that patients with severe Crohn's disease and ulcerative colitis—conditions defined by intractable inflammation—can achieve remission by removing plant-based irritants and shifting to a fat-and-protein-based metabolism?

This gut-health link is likely not just about what is being removed, but what is being produced. We wonder if the production of β-hydroxybutyrate (βHB) serves as a restorative signal for the intestinal barrier. By suppressing the NLRP3 inflammasome, KMT might be providing the gut with the “metabolic silence” it needs to repair itself. This theme is echoed by Newiss, who utilized a carnivore-ketogenic approach to achieve remission in schizophrenia, suggesting that for some individuals, gut-derived inflammation may be the primary driver of neuro-inflammation. The gut, it seems, is not just where we absorb nutrients, but the primary site where our metabolic and immune systems negotiate peace.

## The metabolic mind and the end of silos

The psychiatric evidence in this volume is nothing short of transformative. We see remission in schizoaffective disorder (Laurent, Bellamy, Tague et al.), major depressive disorder (Laurent, Bellamy, Hristova et al.), obsessive-compulsive disorder (MacDonald and Palmer), and bipolar disorder (Schreel et al.). We are left to wonder: have we been misclassifying metabolic brain disorders as purely “psychiatric” for too long?

When a patient experiences “transdiagnostic remission” of PTSD, ADHD, and binge-eating disorder simultaneously through KMT (Bellamy and Laurent), it suggests that these are not separate silos of disease, but different branches of the same metabolic tree. This is further supported by the bibliometric analysis of Yan et al., which highlights the explosive growth in research linking the ketogenic diet to brain health. Whether through improving mitochondrial function or modulating neurotransmitters, KMT appears to offer the brain a more stable and efficient fuel source.

## Expanding the horizon: kidneys and bones

The reach of KMT extends into territories we once thought were purely “structural” or “genetic.” The study on Autosomal Dominant Polycystic Kidney Disease (ADPKD) by Muensterman et al. shows that nutritional ketosis can actually improve renal function and quality of life in this peculiar population of patients with chronic kidney disease (CKD). Similarly, Athinarayanan et al. demonstrate that a remote-care ketogenic intervention can stabilize and even may improve kidney function markers and reduce systemic inflammation in Type 2 Diabetes (T2D) and CKD.

Even our skeletal and reproductive systems appear to be metabolically sensitive. Luo et al. review how βHB influences bone remodeling, suggesting a potential role for KMT in treating osteoporosis.

## Mechanism and the nuances of sex and fertility

As physiologists, we are fascinated by the “under the hood” mechanics revealed in this volume. Eldakhakhny et al. provide evidence that a low-carbohydrate, high-fat diet can modulate autophagy and endoplasmic reticulum (ER) stress, protecting the heart and blood vessels from the damage of metabolic syndrome.

However, we must remain curious about the differences in how we respond. Moss et al. highlight sex-specific metabolic responses, showing that males and females may experience different histone modifications and metabolic shifts in response to KMT. This reminds us that “one size fits all” has no place in the future of metabolic medicine. We must also consider how we monitor these shifts; Fante et al. argue for the importance of rigorous βHB testing to ensure that patients are within the therapeutic windows necessary for these profound physiological changes.

Meanwhile, May et al. explore how fasting and metabolic shifts might influence male fertility, reminding us that every cell in the body is a metabolic actor.

## A condensed conclusion and future perspective

The collective weight of these 19 papers suggests that we are at the dawn of a new era. KMT is no longer just a “weight loss diet”; it is a sophisticated metabolic intervention that can reverse disease processes once thought to be permanent. From the resolution of IBD to the stabilization of psychiatric crises and the protection of renal function, the common denominator is a restoration of cellular energy homeostasis.

The TOWARD study (Buchanan et al.) reminds us of the practical implications: significant weight loss, the deprescription of dozens of medications, and substantial healthcare cost savings. This is a rare “win-win” in medicine.

## Future directions

Looking ahead, we must continue to ask the “uncomfortable” questions with an open mind. We need:

- More research into the carnivore-gut interface to understand why the elimination of plant-based foods facilitates such deep healing in some IBD and psychiatric patients.
- Precision KMT protocols that account for the sex-specific epigenetic changes and individual pharmacokinetic profiles (Nishioka et al.).
- Long-term feasibility studies in diverse populations, such as the Parkinson's study by Worster et al., to ensure these interventions are sustainable.

We invite the scientific community to move forward not with fixed answers, but with the same curiosity that has driven this volume. The metabolic revolution is here, and it is teaching us that the body has a remarkable, untapped capacity for healing when we finally provide it with the right metabolic signals.

